# *UBIAD1* Mutation Alters a Mitochondrial Prenyltransferase to Cause Schnyder Corneal Dystrophy

**DOI:** 10.1371/journal.pone.0010760

**Published:** 2010-05-21

**Authors:** Michael L. Nickerson, Brittany N. Kostiha, Wolfgang Brandt, William Fredericks, Ke-Ping Xu, Fu-Shin Yu, Bert Gold, James Chodosh, Marc Goldberg, Da Wen Lu, Masakazu Yamada, Timo M. Tervo, Richard Grutzmacher, Chris Croasdale, Maria Hoeltzenbein, John Sutphin, S. Bruce Malkowicz, Ludger Wessjohann, Howard S. Kruth, Michael Dean, Jayne S. Weiss

**Affiliations:** 1 Cancer and Inflammation Program, National Cancer Institute, National Institutes of Health, Frederick, Maryland, United States of America; 2 Graduate Partnership Program, National Institutes of Health, Bethesda, Maryland, United States of America; 3 Molecular Medicine Program, Institute for Biomedical Sciences, George Washington University, Washington, D. C., United States of America; 4 Biomedical Science Graduate Program, Hood College, Frederick, Maryland, United States of America; 5 Department of Bioorganic Chemistry, Leibniz Institute of Plant Biochemistry, Halle (Saale), Germany; 6 Division of Urology, Department of Surgery, University of Pennsylvania and Veterans Affairs Medical Center, Philadelphia, Pennsylvania, United States of America; 7 Kresge Eye Institute and Department of Ophthalmology, Wayne State University School of Medicine, Detroit, Michigan, United States of America; 8 Howe Laboratory, Massachusetts Eye and Ear Infirmary, Harvard Medical School, Cambridge, Massachusetts, United States of America; 9 The Eye Institute, Tulsa, Oklahoma, United States of America; 10 Tri-Service General Hospital, Taipei, Taiwan; 11 National Institute of Sensory Organs, National Tokyo Medical Center, Tokyo, Japan; 12 Helsinki University Eye Hospital, Helsinki, Finland; 13 Grutzmacher, Lewis, and Sierra, Sacramento, California, United States of America; 14 Department of Ophthalmology and Visual Sciences, University of Wisconsin School of Medicine and Public Health, Madison, Wisconsin, United States of America; 15 Max-Planck Institut für Molekulare Genetik, Berlin, Germany; 16 Department of Ophthalmology, University of Kansas Medical Center, Prairie Village, Kansas, United States of America; 17 Section of Experimental Atherosclerosis, National Heart, Lung, and Blood Institute, National Institutes of Health, Bethesda, Maryland, United States of America; 18 Kresge Eye Institute and Departments of Ophthalmology and Pathology, Wayne State University School of Medicine, Detroit, Michigan, United States of America; Brigham and Women's Hospital, Harvard Medical School, United States of America

## Abstract

**Background:**

Mutations in a novel gene, *UBIAD1*, were recently found to cause the autosomal dominant eye disease Schnyder corneal dystrophy (SCD). SCD is characterized by an abnormal deposition of cholesterol and phospholipids in the cornea resulting in progressive corneal opacification and visual loss. We characterized lesions in the *UBIAD1* gene in new SCD families and examined protein homology, localization, and structure.

**Methodology/Principal Findings:**

We characterized five novel mutations in the *UBIAD1* gene in ten SCD families, including a first SCD family of Native American ethnicity. Examination of protein homology revealed that SCD altered amino acids which were highly conserved across species. Cell lines were established from patients including keratocytes obtained after corneal transplant surgery and lymphoblastoid cell lines from Epstein-Barr virus immortalized peripheral blood mononuclear cells. These were used to determine the subcellular localization of mutant and wild type protein, and to examine cholesterol metabolite ratios. Immunohistochemistry using antibodies specific for UBIAD1 protein in keratocytes revealed that both wild type and N102S protein were localized sub-cellularly to mitochondria. Analysis of cholesterol metabolites in patient cell line extracts showed no significant alteration in the presence of mutant protein indicating a potentially novel function of the UBIAD1 protein in cholesterol biochemistry. Molecular modeling was used to develop a model of *human* UBIAD1 protein in a membrane and revealed potentially critical roles for amino acids mutated in SCD. Potential primary and secondary substrate binding sites were identified and docking simulations indicated likely substrates including prenyl and phenolic molecules.

**Conclusions/Significance:**

Accumulating evidence from the SCD familial mutation spectrum, protein homology across species, and molecular modeling suggest that protein function is likely down-regulated by SCD mutations. Mitochondrial UBIAD1 protein appears to have a highly conserved function that, at least in humans, is involved in cholesterol metabolism in a novel manner.

## Introduction

Schnyder corneal dystrophy [SCD, MIM 121800] [1], [2] is an autosomal dominant eye disease characterized by an abnormal deposition of cholesterol and phospholipids in the cornea [3], [4]. The resultant bilateral corneal opacification is progressive. Approximately 50% of SCD patients have corneal crystalline deposits [5] which represent cholesterol crystals. Of great interest, two-thirds of affected individuals are hypercholesterolemic [4]. Unaffected individuals in SCD pedigrees may also demonstrate hypercholesterolemia, thus it has been postulated that the corneal disease results from a local metabolic defect of cholesterol processing or transport in the cornea.

A review of 115 affected individuals from 34 SCD families identified by one of the authors (JSW) since 1989, confirmed the finding that the corneal opacification progressed in a predictable pattern dependent on age [5], [6]. All patients demonstrated corneal crystals or haze, or a combination of both findings. While patients have been diagnosed as young as 17 months of age, the diagnosis may be more challenging if crystalline deposits are absent. In acrystalline disease, onset of visible corneal changes may be delayed into the fourth decade [7]. Although many patients maintained surprisingly good visual acuity until middle age, complaints of glare and loss of visual acuity were prominent and increased with age. Disproportionate loss of photopic vision as compared to scotopic vision was postulated to be caused by light scattering by the corneal lipid deposits. Surgical removal of the opacified cornea was reported in 20 of 37 (54%) patients 50 years of age and 10 of 13 (77%) of patients 70 years of age [5].

Recently, several groups described the identification of mutations in *human* SCD patients in a gene with no prior connection to corneal dystrophies or cholesterol metabolism [8]–[12]. The gene, *UBIAD1* (italics is used to indicate the gene), is predicted to encode a membrane protein containing a prenyltransferase domain similar to a bacterial (*E. coli*) protein, UbiA. The *human* gene, *UbiA prenyl-transferase Domain containing 1 (UBIAD1)*, spans 22 kb and the locus gives rise to approximately three different transcripts with up to five unique exons [9]. To date, mutations have been described exclusively in exons 1 and 2, which encode a discrete transcript. Thirty-one apparently unrelated families have been examined and fifteen different mutations have been characterized. Genetic analysis of families revealed a putative mutation hotspot that altered an asparagine at position 102 to a serine reside [11]. Cumulatively, 12/31 (39%) of apparently unrelated families possess this single hotspot alteration. Thus, a major unresolved issue is whether all mutations have similar effect and whether protein activity is up- or down regulated by familial mutations.

The current study examined newly recruited SCD families in order to investigate critical aspects of UBIAD1 protein structure and function. Protein homology across species and conservation of residues mutated in SCD were analyzed. The subcellular localization of UBIAD1 was determined and several forms of cholesterol were quantitated in immortalized peripheral blood mononuclear cell lines derived from SCD patients. Finally, protein threading was utilized to construct a three dimensional model of membrane-bound UBIAD1. The model allowed functional consequences of SCD mutations to be assessed and likely substrates identified that may offer novel therapeutic approaches.

## Results

### Demographics of New SCD Families

**Figure 1:**
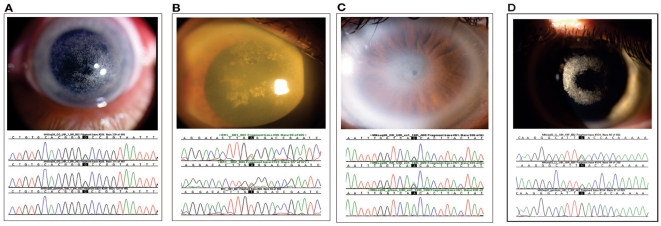
Corneas and *UBIAD1* gene sequencing of SCD probands. Corneal photos (top) and patient sequence chromatograms (bottom) are shown above a wild type sequence. (A) Proband from family GG with a novel A97T mutation. External photograph of the cornea demonstrating central and paracentral crystalline deposition in a 36 year old male. (B) Proband from family AA with a novel V122E mutation. The cornea shows central and paracentral crystalline deposits, diffuse corneal haze, and arcus lipoides in a 69 year old male (top). (C) Proband from family KK with a N102S mutation. The cornea shows central crystalline deposit, mid peripheral haze, and arcus lipoides in a 61 year old male. (D) Proband from family LL with a novel D112N mutation. The cornea shows paracentral crystalline deposition in a 25 year old male.

Ten affected probands with SCD from ten different families were recruited. Six families resided in the United States, Families AA, GG, II, KK, LL, and MM. Four families resided out of the United States: Family CC from Japan, EE from Taiwan, N from Germany, and F1 from Finland. Clinical features of Family N were previously published [13]. No known history of SCD was discovered in five families, EE, GG, II, KK and LL. There was a known family history of SCD in the remaining five families and in three of these families (AA, F1, N) more than one affected individual participated in our study. Affected patients demonstrated findings of SCD including superficial corneal crystals (Figure 1A, top). This study includes a first report of SCD in a family of Native American ancestry and the 69 year old proband demonstrated diffuse cornea haze with scattered superficial crystals and peripheral arcus lipoides (Figure 1B). Probands from other families had similar corneal findings (Figure 1C and 1D).

### Genetic Analysis Yields Novel *UBIAD1* Alterations

Genetic analyses of the *UBIAD1* gene in both new and previously published SCD families are shown with family ethnicities in Table 1 [7]–[11]. Figure 1A–1D (bottom) shows proband sequencing in *UBIAD1* for Families GG, AA, KK, and LL. Five families exhibited novel mutations, A97T (Family GG), D112N (LL), V122E (AA), V122G (F1), and L188H (EE). Five newly analyzed families possessed the same N102S mutation: Families CC, II, KK, MM, and N. Mutations of the N102 residue are shown as distinct in Table 1 but it should be noted that some families may be distantly related and share an N102S mutation due to a founder effect. Over 220 chromosomes from unrelated CEPH individuals were sequenced and examined at the site of each novel mutation. No alterations were found in these healthy individuals confirming that these mutations are likely associated with SCD and not rare polymorphisms.

**Table 1:** Summary of Mutations in SCD Families.^a^

Family	Ethnicity^b^	Gene mutation^c^	Protein^d^	Exon	Loop^e^	Publication
**GG**	**Irish-French Canadian**	**623 G>A**	**A97T**	**1**	**1**	**this report**
F123	Italian	639 A>G	N102S	1	1	7
BB1	British	639 A>G	N102S	1	1	8
BB2	British	639 A>G	N102S	1	1	8
Q	American	639 A>G	N102S	1	1	8
U	American	639 A>G	N102S	1	1	8
Y	German	639 A>G	N102S	1	1	8
1	Irish	639 A>G	N102S	1	1	10
BB	Czechoslavakian	639 A>G	N102S	1	1	9
DD	Chinese-Taiwan	639 A>G	N102S	1	1	9
K	German	639 A>G	N102S	1	1	9
L	American	639 A>G	N102S	1	1	9
R	American	639 A>G	N102S	1	1	9
**CC**	**Japan**	**639 A>G**	**N102S**	**1**	**1**	**this report**
**II**	**American**	**639 A>G**	**N102S**	**1**	**1**	**this report**
**KK**	**American**	**639 A>G**	**N102S**	**1**	**1**	**this report**
**MM**	**American**	**639 A>G**	**N102S**	**1**	**1**	**this report**
**N**	**German**	**639 A>G**	**N102S**	**1**	**1**	**this report** h
**LL**	**French-British-CA** f	**668 G>A**	**D112N**	**1**	**1**	**this report**
F122	Indian	669 A>G	D112G	1	1	7
H	American	687 A>G	D118G	1	1	9
F105	Spanish?-Canadian	689 A>G	R119G	1	1	7
3	African-American	689 A>G	R119G	1	1	10
2	Egyptian	695 C>G	L121V	1	1	10
BB3	British	695 C>T	L121F	1	1	9
O	American	695 C>T	L121F	1	1	9
**AA**	**Native American**	**999 T>A**	**V122E**	**1**	**1**	**this report**
**F1**	**Finnish**	**999 T>G**	**V122G**	**1**	**1**	**this report**
K1	German	845 T>C	S171P	1	2	9
Case 1	Japanese	855 A>G^g^	Y174C	1	2	11
F115	Scottish	858 C>T	T175I	1	2	7
J	Hungarian-American	858 C>T	T175I	1	2	9
T	American	863 G>C	G177R	1	2	8
X	Chinese-Taiwan	863 G>A	G177R	1	2	9
Z	Kosovar	863 G>A	G177R	1	2	9
Case 4	Japanese	876 A>G^g^	K181R	2	2	11
G	German-American	890 G>A	G186R	2	2	9
**EE**	**Chinese-Taiwan**	**897 T>A**	**L188H**	**2**	**2**	**this report**
F118	Canadian	1029 A>G	N232S	2	3	7
Case 6	Japanese	1031 A>C^g^	N233H	2	3	11
FF	African-American	1042 C>G	D236E	2	3	9

a Descending order 5′ to 3′ nucleotide number in the Reference Sequence.

b Ethnicity is given if known, otherwise location of proband is listed.

c Location of mutation in RefSeq NM_013319.2.

d Predicted effect of genetic mutation on protein NP_037451.

e Loop, see Figure 3B.

f CA, Canadian Native American.

g Nucleotides re-numbered based upon updated RefSeq NM_013319.2.

h Clinical description of family N originally described in [14].

A phenotype-genotype discrepancy was noted in Family F1 where a patient was diagnosed as affected by corneal exam but did not possess a V122G mutation. This may be due to difficulties of making the diagnosis of SCD in some patients and/or families [9]. The family and mutation are included in this study as evidence strongly suggests this is a valid SCD alteration. This includes that fact that the same residue was mutated (V122E) in the Family AA proband but not his unaffected sister. Examination of DNA from over 110 healthy individuals failed to indicate the presence of rare polymorphism(s) at this codon.

### Highly Conserved UBIAD1 Residues are Mutated in SCD

**Figure 2:**
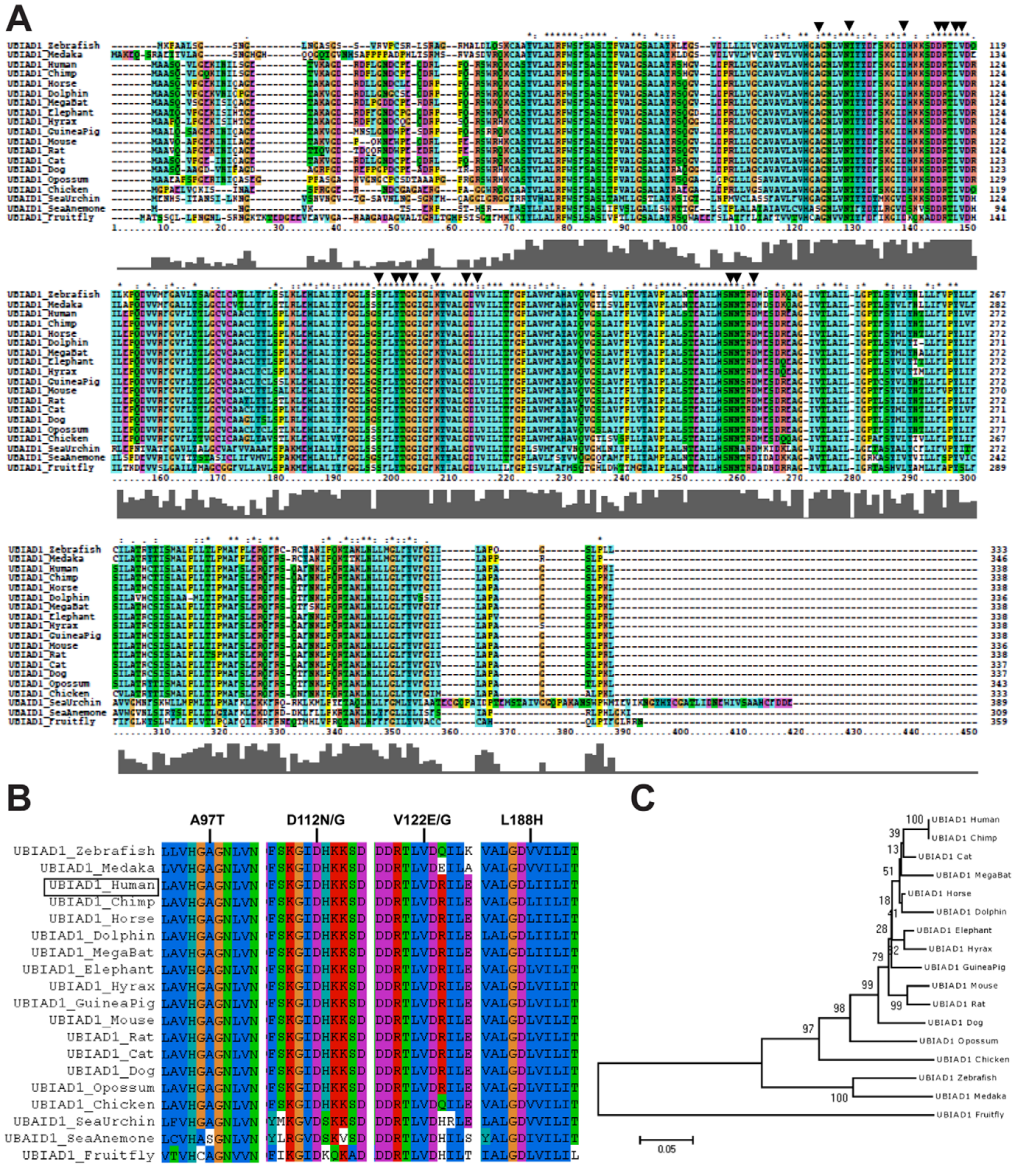
Highly conserved UBIAD1 residues are mutated in SCD. (A) Locations of 17 amino acids mutated in SCD patients are indicated by arrows. Taller bars in the graph below the alignment indicate greater conservation. (B) Regions of alignment encompassing *human* SCD mutations: A97, D112, V122, L188, are shown. The position of the *human* protein in the alignment is indicated on the left (box). (C) Evolutionary relationships based upon UBIAD1 homology.

The entire protein sequence was examined across species, with a focus on residues mutated in SCD because mutation of conserved residues could suggest interruption of an ancient function. A previous analysis, examined homology across 23 amino acids of a putative prenyltransferase active site [11], [14]. UBIAD1 homologs were identified in 19 species and protein sequences were aligned using ClustalX [15] (Figure 2) Homology was high among mammals based upon pairwise alignment scores. Compared to human, chimp was 99.7% similar, mouse 92%, rat (92%), cattle (91%), and dog (89%). The protein was generally conserved in non-mammalian vertebrates. Compared to human, similarity to chicken was 81.7%, zebrafish 78.9%, and fruitfly 59.6%.

Locations of 17 amino acids mutated in SCD are indicated (Figure 2A, 2B). Fifteen out of 17 (88%) were universally conserved in all 19 organisms examined from sea urchins to humans, including A97, D112, V122, L188. Groups of SCD mutations were clustered in regions of protein exhibiting the highest degree of conservation between species. These were separated by regions of protein that are less conserved. Based upon the alignment, a phylogenic tree was created (Figure 2C). The tree is consistent with the high conservation of the protein in mammalian species and lesser but substantial conservation in other vertebrates.

### Linear and 2D Protein Models

**Figure 3:**
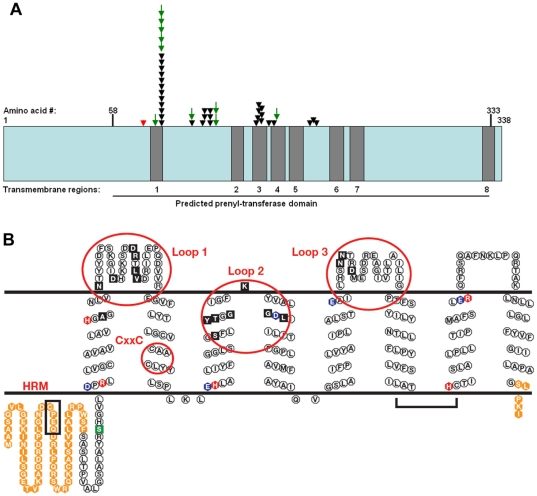
Locations of familial SCD alterations in UBIAD1. (A) A linear diagram of the UBIAD1 protein with independent familial mutations (arrows). Novel familial mutations presented in this study are indicated by green arrows. Previously published SCD mutations are indicated (black arrows). Predicted transmembrane (grey boxes) and prenyltransferase domains (horizontal line, bottom) are indicated. A previously described S75F SNP is indicated (red arrow). Adapted from Ref. 11. (B) Locations of SCD mutations in a proposed 2-D model of UBIAD1 in a lipid bilayer. Solid Black: SCD mutations, Orange: amino acids outside the prenyltransferase domain, Blue: acidic residues, Red: basic residues, HRM: heme regulatory motif (box), CxxC: oxidoreductase motif (CAAC, small circle), Green: S75F polymorphism.

A linear diagram and 2-D model of UBIAD1 in a lipid membrane (Figures 3A and 3B) demonstrate the number and location of newly reported and previously published familial SCD mutations [8]–[12]. Family GG possessed the most N-terminal SCD alteration yet described, A97T. N102S, the site of 17 familial mutations (41% of families), is located at the first transmembrane spanning region. The mutated amino acids (Figure 3B) occur in regions of the protein on one side of the membrane (Loops 1–3). Loop 1 of the protein is affected by 9/10 of these newly reported mutations. Other mutations described in this study, D112N and two alterations at V122 (V122E and V122G), appear to affect aqueous portions of Loop 1. The single Loop 2 mutation is L188H which extends this cluster of mutations towards the C-terminus.

### Localization of UBIAD1 to Mitochondria in Keratocytes

**Figure 4:**
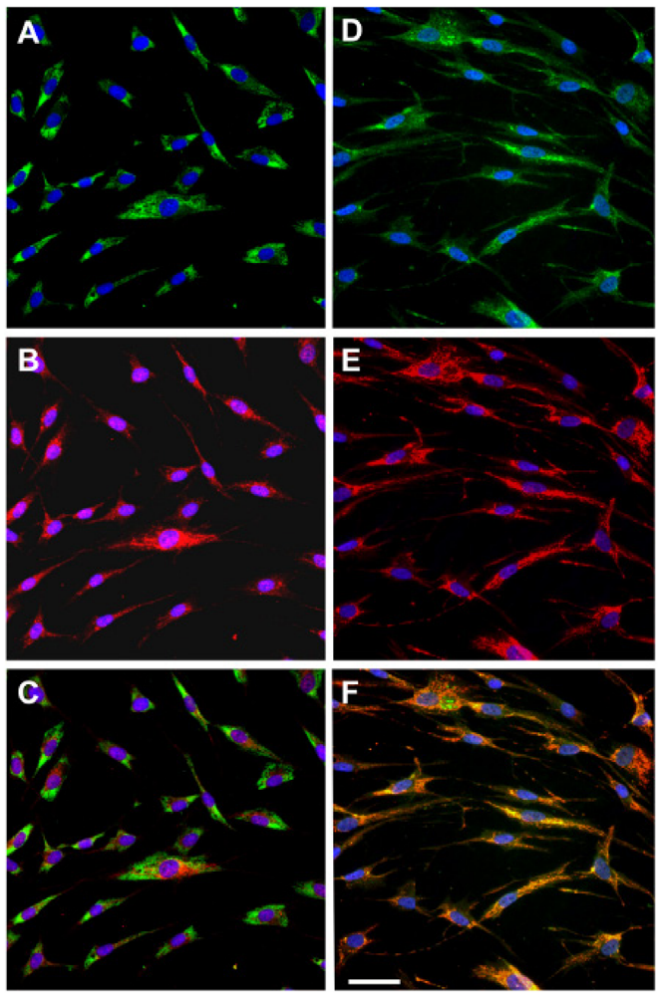
Cellular localization of wild type *human* UBIAD1. Co-localization within cultured normal *human* keratocytes of UBIAD1 protein and protein disulfide isomerase, an enzyme in endoplasmic reticulum, is shown in panels A–C. Co-localization of UBIAD1 and OXPHOS complex I, an enzyme in mitochondria, is shown in D–F. UBIAD1 labeling is red (B and E). Protein disulfide isomerase and OXPHOS I are green (A and D). UBIAD1 did not co-localize with the endoplasmic reticulum (C), but did co-localize with mitochondria (co-localizing red and green show as orange in F). Bar is 50 µm and applies to all.

**Figure 5:**
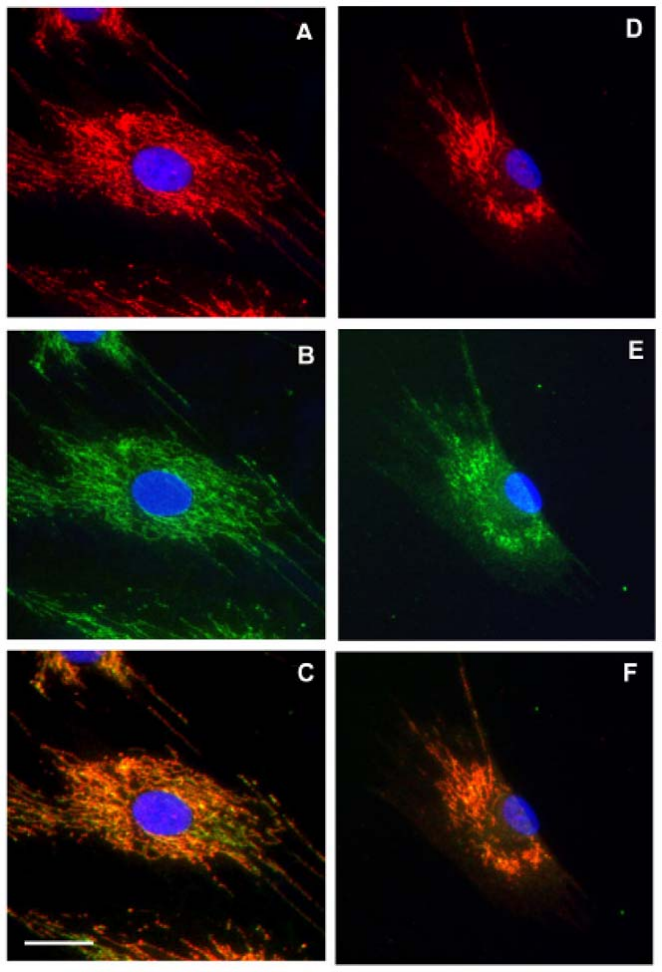
Localization of SCD mutant UBIAD1. Co-localization of UBIAD1 and OXPHOS complex I mitochondrial marker in keratocytes derived from the Family KK proband (N102S mutation, panels A–C) and a healthy donor (D–F). UBIAD1 (red, A and D) and a mitochondrial marker (green, B and E) show co-localization (orange) in both normal (F) and SCD disease keratocytes (C). Bar is 25 µm and applies to all.

To examine whether SCD mutations altered UBIAD1 protein trafficking, the subcellular localization of wild type and mutant *human* UBIAD1 was examined (Figure 4). Localization within cultured normal *human* keratocytes of UBIAD1 and protein disulfide isomerase, an enzyme marker for the endoplasmic reticulum, is shown (Figure 4A–4C). Co-localization of UBIAD1 and a subunit of OXPHOS complex I (NADH dehydrogenase), an enzyme in mitochondria, is shown in Figure 4D–F. UBIAD1 did not co-localize with the endoplasmic reticulum (Figure 4C), but did co-localize with mitochondria (orange in Figure 4F). Figure 5 presents localization of UBIAD1 in SCD and normal keratocytes. Co-localization of SCD mutant UBIAD1 protein and OXPHOS complex I mitochondrial marker in disease keratocytes (Figure 5A–5C) and normal *human* keratocytes (Figure 5D–F) is shown.

### Analysis of Cholesterol in SCD Patients

Mutation of *UBIAD*1 in SCD is thought to result in deregulation of cholesterol and lipid metabolism, resulting in an abnormal accumulation of these substances in the cornea leading to corneal opacification and visual loss [5]. No significant differences were observed in total cholesterol, cholesteryl ester, and unesterified cholesterol in SCD and healthy patient B cell lines (Table 2).

**Table 2:** Analysis of Cholesterol in SCD and Healthy Patients.

Patient B cell	SCD status	TC (nmol/mg pr.)^a^			UC (nmol/mg pr.)^b^			CE (nmol/mg pr.)^c^			Ester/Total (%)		
line ID		AVE	±	SD	AVE	±	SD	AVE	±	SD	AVE	±	SD
66K00597	**unaffected control**	**44.0**	**±**	**8.1**	**44.5**	**±**	**8.7**	**−0.6**	±	**2.2**	**−1.2**	±	**4.5**
BUC692RDP692A													
66K00594	**unaffected control**	**51.7**	**±**	**8.4**	**49.9**	±	**8.6**	**1.8**	±	**0.2**	**3.5**	±	**0.9**
BUC704RDP704A													
66K00595	**unaffected control**	**57.5**	**±**	**0.3**	**55.1**	±	**0.8**	**2.4**	±	**1.0**	**4.2**	±	**1.8**
BUC708RDP708A													
64101374	**affected proband**	**44.1**	±	**0.7**	**42.2**	±	**1.3**	**1.8**	±	**0.6**	**4.2**	±	**1.4**
	**SCD Family AA**												
64101375	**unaffected sibling**	**46.9**	**±**	**2.3**	**45.9**	±	**2.2**	**1.0**	±	**0.2**	**2.2**	±	**0.4**
	**SCD Family AA**												
64101376	**affected proband**	**51.1**	**±**	**1.5**	**49.6**	±	**1.2**	**1.5**	±	**0.3**	**3.0**	±	**0.5**
	**SCD Family GG**												

a TC: total cholesterol.

b UC: unesterified cholesterol.

c CE: cholesteryl ester.

### Protein Threading Model of UBIAD1 Protein

**Figure 6:**
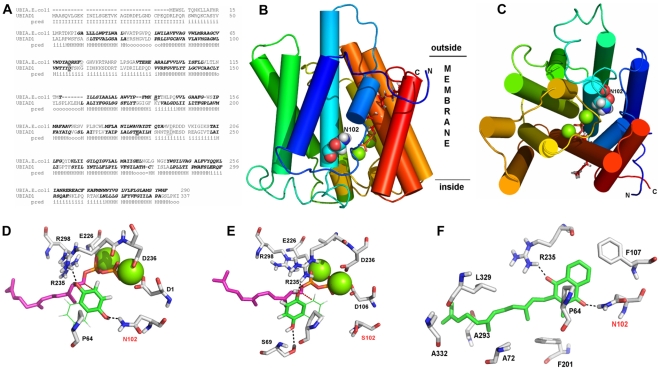
Three dimensional modeling of *human* UBIAD1. (A) Alignment of *E.coli* UbiA and *human* UBIAD1 with predicted transmembrane helices (pred, bold italics), H = helix, I = inside, o = outside. Organic diphosphate binding residies are underlined. (B) Rainbow representation (side view) of a putative 3D-structure of UBIAD1 in the membrane. Approximate location of the lipid bilayer is indicated (horizontal lines). Inside and outside are arbitrary labels of membrane sidedness. Green spheres represent magnesium cations in the active site with a docked farnesyl-diphosphate (red stick representation). The side chain of N102 is shown as a space-filled atom. (C) Top view as described in Fig. 6B. Magenta atoms show potential binding of a putative substrate. (D) Hypothetical docking of farnesyldiphosphate and a 1,4-dihydroxy aryl compound. Substrate recognition by N102 (arrow) and R235 via hydrogen bonds and by hydrophobic interactions with P64 are indicated (dashed lines). The distance of the C2-atom of the hydroquinone to the C1-atom of the farnesyl moiety is 3.8 Å (red dashed line). (E) Docking arrangement of the two putative substrates as in Figure 6D upon *in silico* mutation of UBIAD1 from asparagine 102 to serine (arrow). The aromatic substrate is no longer recognized by N102, but by S69 and, as before, by R235 and P64. C2 of the aromatic substrate is no longer positioned correctly to allow prenylation. (F) Active site of UBIAD1 with a menaquinone-farnesyl derivative that optimally docks to the protein. Substrates with longer fatty acid tails were also successfully docked. The interaction is stabilized by hydrogen bonds (dashed lines) with N102 and R235. R235 may be influenced by neighboring residues, N232, N233, and D236, which cause SCD when altered. The quinone moiety and farnesyl chain are recognized by P64, F107, and other indicated residues via hydrophobic interactions.

To examine UBIAD1 structure-function relationships and assess the potential impact of SCD mutations, three dimensional (3D) modeling was performed using protein threading. Available X-ray structures of prenyl-converting enzymes were examined, including a recently developed model of the all-alpha-helical *E. coli* UbiA [16]–[19]. Modeling using the Molecular Operating Environment (MOE) indicated UbiA but not other proteins possessed an arrangement of alpha helical structural elements that could be superimposed on UBIAD1 (Figure 6A). The positional placement of geranylpyrophosphate and a single magnesium cation were extracted from the *E. coli* UbiA model and fitted into the model of UBIAD1. A second magnesium cation was manually added to the model due to an additional aspartate close to the putative binding site of the pyrophosphate moiety in UBIAD1.

PROCHECK assessment of stereochemical quality of the model obtained from MOE and refined using YASARA indicated that 86.7% of all amino acid residues were located in the most favored area and only three residues were in disallowed (uncertain) loop regions. All parameters evaluated were better (overall G-factor) than similar values for an analogous X-ray crystal structure at a 2 Å resolution. Inspection of the fold quality revealed a quality indication of 94%, with low quality scores in only five small regions. Over 30 models were generated and evaluated to obtain the model shown in Figure 6B and 6C. Transmembrane helices created an approximate circular pattern to form a substrate binding cleft on one side of the membrane. The N102 residue occupied a position where the first TM helix exited the membrane and its sidechain pointed inwards towards the center of a putative prenyldiphosphate binding pocket (Figure 6C). A docked farnesyldiphosphate is shown with magnesium cations in the active site. The prenyl substrate appeared to approach the active site containing N102 from the central cavity.

The model allowed docking of potential ligand(s)/substrate(s) to be examined including those involved in reactions catalyzed by UbiA (Figure S1) [16], [17]. Database searches revealed homology between the binding site in the model and 1,4-dihydroxy-2-naphthoate octaprenyltransferases (e.g. Q17BA9_AEDAE). This suggested that similar to aromatic prenyltransferases a second (aromatic?) substrate may be involved in catalysis, e.g. 4-hydroxybenzoate (cf. UbiA) or a 1,4-dihydroxy-naphthaline derivative. For the latter case, bacterial menaquinone (vitamin K-2) is the product of a similar prenylation reaction, a farnesylation in position 3 of the aromatic substrate 2-methyl-1,4-naphthohydroquinone. This molecule is structurally similar to 1,4-dihydroxy-2-naphthoate. Speculative second substrates 4-hydroxybenzoic acid and 1,4-naphthalin-diol were successfully docked into the putative active site of the model (Figure S2). A tertiary model showed naphthalinediol docked preferentially in the central cavity in close proximity to amino acid, N102.

Docking simulations were compared using models of wild type and SCD mutant (N102S) UBIAD1 protein and substrates, farnesyldiphosphate and a 1,4-dihydroxy aryl compound (Figure 6D and 6E). The diphosphate binding site was identified in the putative active site of both models in close proximity to N102. In the wild type protein, N102 formed weak hydrogen bonds to the 1,4-dihydroxy aryl compound (dotted line) which were lost upon mutation to a serine residue. Lastly, substrate docking was examined using prenylated aromatics with a role in *human* metabolism. This showed that menaquinone (vitamin K) fits excellently into the substrate binding cleft of UBIAD1 models (Figure 6F). While this class of molecules is not a likely substrate for UBIAD1, they may be ligands that are transferred to protein binding partners.

**Figure 7:**
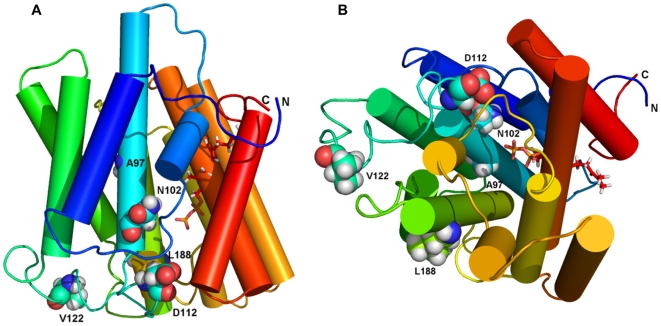
Locations of selected SCD alterations. (A) Sideview of UBIAD1 showing locations of wild type amino acids mutated in SCD. (B) Top view as in Figure 7A. In each view, only several residues mutated in SCD are visible. Farnesyldiphosphate is shown as a stick representation. The sidechains of SCD mutations reported in this paper are shown as spacefilled atoms: A97, N102, D112, V122, and L188.

Amino acids mutated in SCD were found in the vicinity of the active site, including A97, N102, D112, V122, and L188. Figure 7A, 7B, S2, and S3 show side and top views, respectively. The N102 residue is shown as a spacefill atom with a docked farnesyldiphosphate. Significantly, a previously described polymorphism, S75F [8], [9], was not identified as functionally important to substrate docking or catalysis.

## Discussion

Recruitment and analysis of new families with SCD continues to facilitate investigation of the genotypic spectrum of this disease. Of ten new families recruited for this study, five possessed novel *UBIAD1* alterations. SCD mutations A97T (Family GG), V122E (Family AA), and V122G (Family F1) expand the size of the Loop 1 mutation cluster (Figure 3B). Similarly, L188H (Family EE) expands the Loop 2 cluster. All five of the novel amino acid substitutions represent non-conservative changes that are consistent with previously described alterations (Table 1): A97T (nonpolar to polar), D112N (negative to neutral), V122E (nonpolar to polar-negative), V122G (aliphatic to non-aliphatic), and L188H (nonpolar to polar-positive).

Three distinct lines of evidence indicate that SCD results from loss of function of UBIAD1 protein due to a mutation: genetics, experimental mutagenesis of UbiA, and modeling of substrate-UBIAD1 interactions. There are three cases (Table 1) where UBIAD1 amino acids were mutated to other residues in SCD families, aspartic acid 112 to an asparagine or a glycine, leucine 121 to a valine or a phenylalanine, and valine 122 to a glutamic acid or a glycine. There were significant chemical differences between resulting mutant amino acids and from wild type. For example, substitution of non-polar valine 122 with either polar, negative glutamic acid or non-polar, neutral glycine results in SCD. This suggests that loss of valine 122 may be necessary for the formation of SCD rather than a gain of function.

Despite low overall homology between *E. coli* UbiA and *human* UBIAD1 proteins, comparison of individual amino acids aligned in Figure 6A suggest SCD mutations may result in loss of function of UBIAD1. In prior work on UbiA [16]–[18], [20], five aspartic acid residues were judged as crucial for catalytic activity based on modeling. These were individually mutated and all five inhibited product formation by >95%. Figure 6A shows that mutagenized UbiA residues (R137 and D191) aligned with amino acids in UBIAD1 that are mutated in *human* SCD, L181 and D236. Thus, mutagenized UbiA amino acids that resulted in loss of function aligned to UBIAD1 residues mutated in SCD. This is a second piece of evidence that a SCD mutation may lead to loss of function of UBIAD1.

Modeling of UBIAD1 substrate docking indicates critical roles for several residues mutated in SCD by suggesting a mutation of these residues would block critical steps in catalysis. For example, naphthalin-1,4-diol was docked as a speculative second substrate of UBIAD1 and fitted nicely into the binding pocket (Figure S2). However, a SCD mutation, N102S, changed binding of this substrate completely, rendering its prenylation at position 3 impossible (Figure 6D and 6E). Although detailed modeling of active site residues is full of uncertainty, this approach is supported by modeling of UbiA that predicted the loss of enzyme activity and was experimentally verified [16]. Further, modeling of UbiA was able to connect the decreases in enzyme activity to specific chemical functions of mutated residues, i.e. activation of a phenolate intermediate by D191. This residue aligned to SCD mutation D236 in the UBIAD1 protein. This provides a third indication that a mutation of UBIAD1 in SCD may be due to a loss of function of the protein/enzyme.

The result that UBIAD1 did not localize with a marker for endoplasmic reticulum (Figure 4) while wild type and N102S mutant UBIAD1 did co-localize with a mitochondrial marker, OXPHOS complex (Figure 4F), demonstrates that mislocalization of N102S mutant protein is not a factor in SCD. Mitochondrial localization is surprising in light of a previous report demonstrating interaction between UBIAD1 (also known as TERE1) and apolipoprotein E [21], [22]. To our knowledge, a mitochondrial localization for apolipoprotein E has not been reported. However, some UBIAD1 immunostaining was localized outside of mitochondria in these analyses making interaction with apolipoprotein E outside of mitochondria possible.

SCD has been associated with deregulation of cholesterol metabolism in the cornea as well as systemic hypercholesterolemia [3]. The *UBIAD1* gene had been shown to be expressed in B-cells [20], however we found no significant differences in levels of cholesterol metabolites in extracts of B-cell lines established from SCD patients compared to an unaffected family member and healthy donors (Table 2). This may indicate that UBIAD1 has a specialized corneal function, perhaps relying on specific protein-protein interactions (such as with apolipoprotein E) or in post-translational modification of binding partners, perhaps with a cholesterol or cholesterol-like moiety [23]. In this regards, substrate docking simulations indicated that menaquinone fits well into the interior of UBIAD1 (Figure 6F). This may be significant since a relationship between menaquinone and cholesterol metabolism has been suggested by prior publications [24].

Experiments to determine if UBIAD1 will accept oligoprenyl diphosphates as a substrate or ligand may be informative, but the 3D protein model clearly shows an optimal binding pocket for this type of compound (Figure 6B and 6C). UBIAD1 may be an aromatic prenyl transferase as indicated by its closest known protein homologues. If so, a second substrate or ligand moiety may be involved in enzyme catalysis, e.g. 4-hydroxy benzoate or a 1,4-dihydroxy-naphthaline derivative.

The high degree of conservation of the protein across species, and particularly residues mutated in SCD, indicates that the protein may have an essential or at least ancient metabolic function. These function(s) may play a role outside the cornea as the gene is widely expressed in *human* tissues [20] and the protein is present in species without eyes such as the sea urchin (Figure 2). Modeling of UBIAD1 indicates the possibility of aromatic prenylation as an enzyme activity. This biochemistry may evolutionarily be at least as old as aerobic life, and it has been described in *human* metabolism. Accordingly, UBIAD1 may have a common origin directly from *E. coli* UbiA, but may not necessarily act as a transferase (see Figure S1).

Presently, the only treatment for SCD is corneal replacement by penetrating keratoplasty (PKP) once corneal opacification causes decreased vision. PKP is performed in the majority of patients above the age of 50 years with SCD [5]. Unfortunately, there are no current therapies to prevent the progressive lipid deposition in the cornea which results in this visual loss.

Prior studies have demonstrated that normalizing blood cholesterol levels does not affect the relentless deposition of corneal lipid that occurs with age [25]. Hopefully further understanding about the impact of *UBIAD1* gene mutations in SCD will potentially lead to interventional strategies to prevent the relentless accumulation of corneal lipid which results in visual loss in these patients. Our results suggest that UBIAD1 protein function is lost or decreased by SCD mutations. Thus, therapeutic analogs of substrates which were successfully docked to the UBIAD1 model (Figure S4) may further inhibit rather than restore protein function. Examination of protein binding partners may allow useful therapeutic targets to be identified.

## Materials and Methods

### Patients and Samples

New patients were recruited as previously described under Institutional Review Board approval of University of Massachusetts Medical Center and Wayne State University School of Medicine [5]. IRB approval was also obtained from the NIH Office of Human Subjects Research. Creation of cell lines and analysis of patient samples described in this study are covered by the IRB approved protocol. All adults and parents of minors who participated in the study provided written informed consent under the research tenets of the Declaration of Helsinki. Affected probands were recruited from other physicians and also were self recruited by internet contact with JSW. Family history, ophthalmologic examination, blood samples were obtained on all affected patients. When possible, other family members were recruited to confirm the inherited nature of the SCD mutations. Ophthalmologic examination included assessment of visual acuity and slit lamp examination of the cornea detailing location and characteristics of the corneal opacity. Notation was made as to the presence of central corneal opacity, mid peripheral haze, arcus lipoides and corneal crystalline deposition. Slit lamp photographs were obtained whenever possible to document the diagnosis.

### DNA Extraction and Sequencing

DNA was extracted using standard methods and either Puregene (Gentra/Qiagen, Valencia, CA) or other Qiagen reagents (All Prep DNA/RNA Kit). Genetic analysis of patient DNA was performed as previously described, [8]–[10] except that FastStart PCR reagents (Roche, South San Francisco, CA) and ABI (Foster City, CA) thermal cyclers were used. Sanger sequencing was performed using Big Dye reagents (ABI) and subjected to chromatography using a 3730 Genetic Analyzer (ABI). Sequence chromatograms were analyzed using Sequencher, v4.8 (GeneCodes, Ann Arbor, MI). Over 100 control DNAs from healthy donors were examined by double stranded sequencing for each mutation to insure that mutations were novel, associated with SCD, and unlikely to be rare polymorphisms. Healthy DNA samples were obtained from the Dean Lab database (MD) and the Coriell Institute for Medical Research (Camden, NJ).

### Homology and Phylogeny

The following UBIAD1 sequences from 19 indicated species were identified using the Ensembl database: NP_037451.1 [*Homo sapiens*, human], XP_001137312.1 Predicted [*Pan troglodytes*, chimp], XP_544571.1 Predicted [*Canis familiaris*, canine], XP_585287.3 Predicted [*Bos taurus*, cattle], XP_001492378.1 Predicted [*Equus caballus*, horse], NP_082149.1 [*Mus musculus*, mouse], XP_233672.1 Predicted [*Rattus norvegicus*, rat], NP_001026050.1 [*Gallus gallus*, chicken], XP_686705.2 Predicted [*Danio rerio*, zebrafish], ENSORLT00000000192 Predicted [*Oryzias latipes*, medaka/killifish], NP_523581.1 [*Drosophila melanogaster*, fruitfly], XP_001639930.1 Predicted [*Nematostella vectensis*, sea anemone], XP_001175897.1 Predicted [*Strongylocentrotus purpuratus*, sea urchin], ENSCPOG00000011678 Predicted [*Cavia porcellus*, Guinea pig], ENSFCAG00000000057 Predicted [*Felis catus*, cat], ENSLAFG00000015673 Predicted [*Loxodonta Africana*, African elephant], ENSPCAG00000003043 Predicted [*Procavia capensis*, hyrax], ENSMODG00000011080 Predicted [*Monodelphis domestica*, opposum], ENSTTRG00000001324 Predicted [*Tursiops truncates*, bottlenose dolphin], ENSPVAG00000014788 Predicted [*Pteropus vampyrus*, megabat/flying fox]. Alignments were performed using Clustal 2.0.11 [15]. A global alignment performed on all proteins was followed by local optimization of overlapping, sequential regions of protein in approximately fifty amino acid increments.

### Localization of *Human* UBIAD1

Normal *human* keratocytes were purchased from ScienCell Research Lab (Carlsbad, CA). Schnyder corneal dystrophy and normal *human* keratocytes were cultured at 37°C in Fibroblast Medium (catalogue no. 2301) also obtained from ScienCell Research Lab. For immunofluorescence labeling experiments, the keratocytes were rinsed three times with DPBS before fixing with 2% formaldehyde for 10 minutes at room temperature. Cells were then blocked with 10% FBS in DPBS (FBS blocking solution) for 30 minutes, and then treated 15 minutes with avidin/biotin blocker (Vector Laboratories, Burlingame, CA) with a DPBS rinse between each step of the procedure described by the manufacturer. Chicken anti-UBIAD1 was diluted to 5 µg/ml in FBS blocking solution containing 0.2% Triton X-100, and incubated with keratocytes for one hour at room temperature. After three five-minute rinses with DPBS, biotinylated goat anti-chicken IgY (catalogue no. 103-065-155 from Jackson Immunoresearch, West Grove, PA) diluted to 5 µg/ml in FBS blocking solution was incubated with keratocytes for one hour. This primary labeling of UBIAD1 protein was then visualized by incubating keratocytes with 5 µg/ml Alexa 594 (red) streptavidin diluted in DPBS (catalog no. S32356, Molecular Probes, Eugene, Oregon).

To determine the subcellular localization of UBIAD1, keratocytes were further incubated one hour with either 5 µg/ml mouse IgG2b monoclonal anti-protein disulfide isomerase (catalogue no. S34253, Molecular Probes), an endoplasmic reticulum marker; or 5 µg/ml mouse IgG1 monoclonal anti-OXPHOS Complex I subunit, NADH dehydrogenase (catalogue no. A31857, Molecular Probes), a mitochondrial marker. This was followed by incubation with 5 µg/ml Alexa fluor 488 (green) anti-mouse IgG (catalogue no. A11029, Molecular Probes) for one hour to label the subcellular markers. All antibodies were diluted in FBS blocking solution.

### Cholesterol Measurements

Lymphocytes were isolated from patient blood samples using lymphocyte separation medium and were immortalized using Epstein-Barr virus. Standard culture conditions utilized RPMI 1640 media (Invitrogen), 15% fetal bovine serum (Hyclone, Waltham, MA), and 2× L-glutamine (Invitrogen). Six well plates were used to grow approximately 1 million cells per well, which were rinsed three times each with Dulbecco's phosphate-buffered saline (DPBS) plus Mg^2+^, Ca^2+^, and 0.2% bovine serum albumin (BSA), and then DPBS plus Mg^2+^ and Ca^2+^. Cells were harvested from wells by scraping into 1 ml of distilled water, and then processed as described previously [26]. Lipids were extracted from an aliquot of cell suspension using the Folch method [27]. The cholesterol content of cells was determined according to the fluorometric method of Gamble *et al.* [28]. Protein content was determined on another aliquot of cell suspension by the method of Lowry *et al.* using BSA as a standard [29].

### Protein Models

UBIAD1 transmembrane helices and topology were analyzed using the HMMTOP program and server [30], [31] The Brookhaven Protein Data Bank (PDB) and PHYRE (**P**rotein **H**omology/analog**Y** **R**ecognition **E**ngine) were searched for proteins homologous to UBIAD1 using BLASTp [31]. Homology between UBIAD1 and other prenyltransferases was examined using MOE (Molecular Operating Environment, Chemical Computing Group Inc., Montreal, Canada). Transmembrane helices were manually examined by using available X-ray structures of prenyl converting enzymes as templates, such as prenyl synthases (cyclases), protein prenyl transferases, and the recently developed model of the all-alpha-helical *E. coli* UbiA prenyltransferase [16], [17]. Alignment was performed as previously described [16]. The positional placement of geranylpyrophosphate and a single magnesium cation were extracted from the *E. coli* UbiA model. The model obtained from MOE was refined using the molecular dynamics refinement tool YASARA and stereochemical quality was analyzed with PROCHECK [32]. All parameters evaluated were better (overall G-factor) then required for an analogous X-ray of better than 2 Å resolution. Inspection of the fold quality was done with ERRAT [33].

Substrate suitability was approached by examining homologous proteins in the Uniprot Knowledgebase Release 15.2 database. Substrates examined are available upon request. Substrate binding and dynamics (4-hydroxybenzoic acid and 1, 4-naphthalin-diol) were evaluated using automated docking and molecular dynamics simulations (GOLD [34]).

### Web Resources

The URLs for data presented herein are as follows: Online Mendelian Inheritance in Man (OMIM), http://www.ncbi.nlm.nih.gov/OMIM/; Entrez Nucleotide, http://www.ncbi.nlm.nih.gov/nuccore; Ensembl, http://www.ensembl.org/index.html; HMMTOP, http://www.enzim.hu/hmmtop/index.html; PHYRE, http://www.sbg.bio.ic.ac.uk/~phyre; RCSB Protein Data Bank, http://www.rcsb.org/pdb/home/home.do; BLASTp, http://blast.ncbi.nlm.nih.gov/Blast.cgi; Molecular Operating Environment, http://www.chemcomp.com; YASARA, http://www.yasara.org; PROCHECK, http://www.biochem.ucl.ac.uk/~roman/procheck/procheck.html; ERRAT, http://nihserver.mbi.ucla.edu/ERRATv2/; UniProt knowledge base, http://www.expasy.ch/sprot/; TOPO2, http://www.sacs.ucsf.edu/TOPO2/.

## Supporting Information

Figure S1 Key enzymatic prenylation reaction catalyzed by UbiA during biosynthesis of ubiquinone [16], [17]. Prenylation of 4-hydroxybenzoic acid by oligoprenyl diphosphates are shown (n>1). A two substrate reaction is shown similar to that proposed for human UBIAD1 (see Discussion).
(1.19 MB TIF)

Figure S2 Docking simulation with naphthalinediol as a putative substrate. Tertiary protein structure model of human UBIAD1 with eight transmembrane helices and a putative naphthalinediol substrate docked (shown as a spacefill atom representation).
(2.99 MB TIF)

Figure S3 Models showing locations of Loops 1–3 containing clusters of SCD mutations. See also Figure 3B for comparison, to identify SCD mutations in each loop. Two views are shown, a side view (left side) and top view (right side). These highlight the loop regions containing amino acids implicated in SCD. Loop 1 (containing amino acids A97 to R132) is shown in orange, loop 2 (Y174 to A184) in blue, and loop 3 (L229 to S257) in green. Mutated S102 is shown as a spacefill atom and a docked farnesyldiphosphate is shown as a stick representation (red).
(6.59 MB TIF)

Figure S4 Structures of potential substrates successfully docked with the UBIAD1 model. (A) Farneslydiphosphate (C15H25O7P2-3). (B) Menaquinone (C11H8O2). (C) Naphthalenediol (C10H8O2).
(0.93 MB TIF)
