# Supplementary figures and images for: UBIAD1 Mutation Alters a Mitochondrial Prenyltransferase to Cause Schnyder Corneal Dystrophy
Source: PLoS One. 2010 May 21;5(5):e10760. doi: 10.1371/journal.pone.0010760 (PMC2874009; doi:10.1371/journal.pone.0010760)

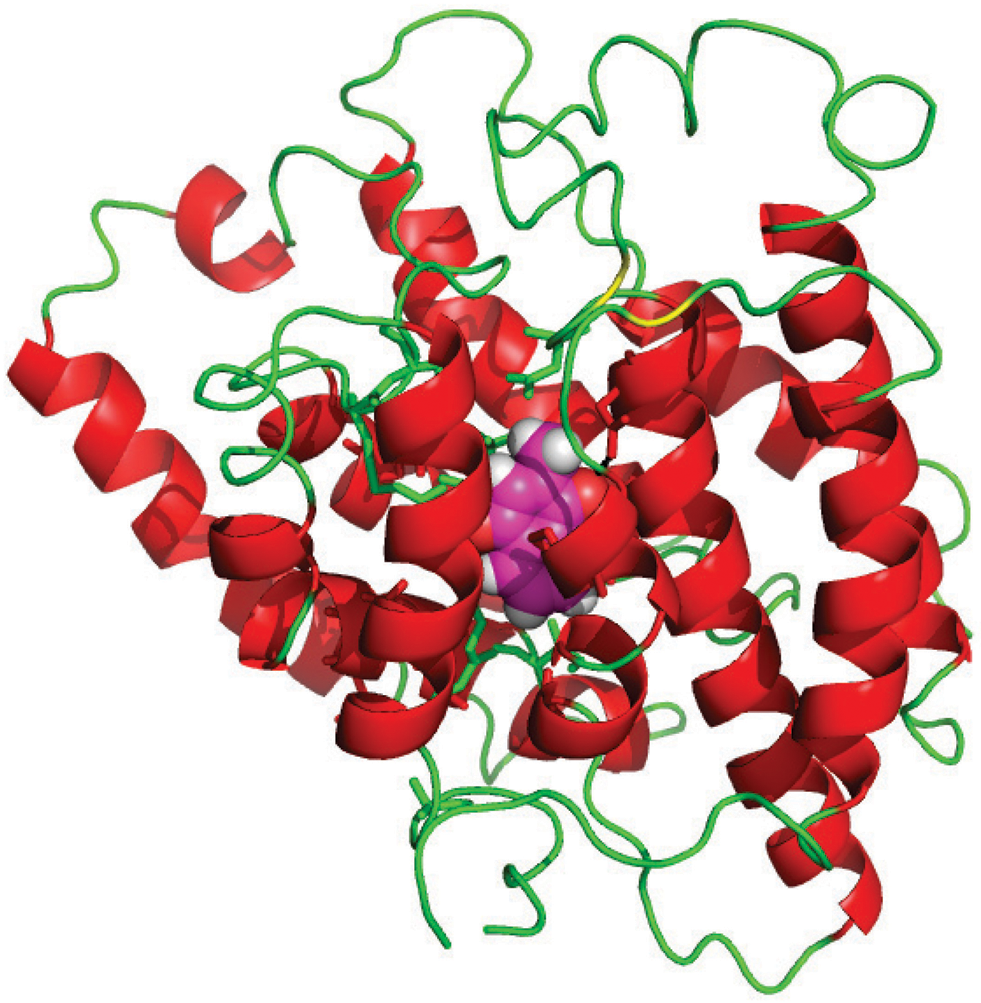

Supplement: Figure S2 — Docking simulation with naphthalinediol as a putative substrate. Tertiary protein structure model of human UBIAD1 with eight transmembrane helices and a putative naphthalinediol substrate docked (shown as a spacefill atom representation). (2.99 MB TIF) [file pone.0010760.s002.tif]

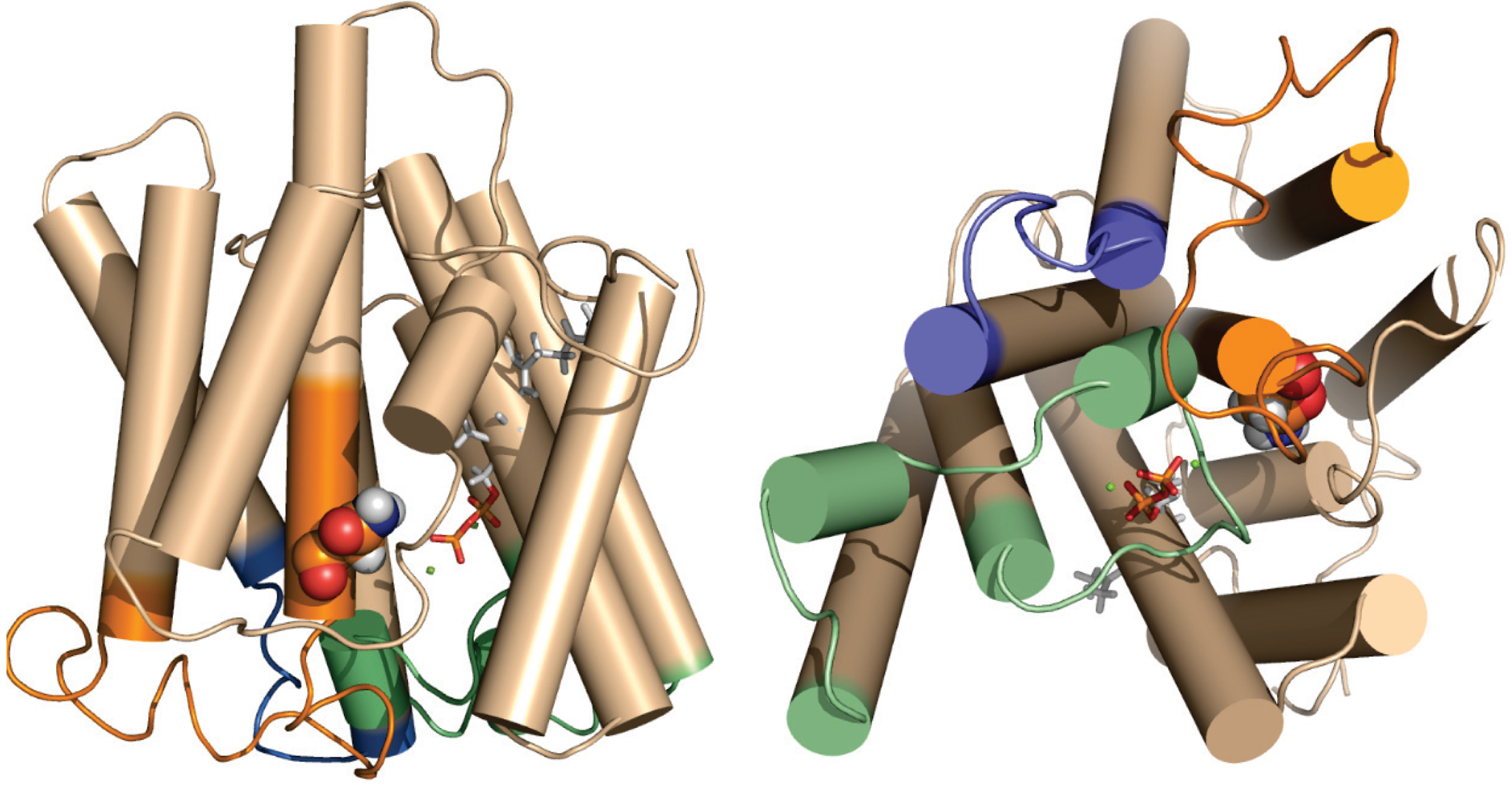

Supplement: Figure S3 — Models showing locations of Loops 1–3 containing clusters of SCD mutations. See also Figure 3B for comparison, to identify SCD mutations in each loop. Two views are shown, a side view (left side) and top view (right side). These highlight the loop regions containing amino acids implicated in SCD. Loop 1 (containing amino acids A97 to R132) is shown in orange, loop 2 (Y174 to A184) in blue, and loop 3 (L229 to S257) in green. Mutated S102 is shown as a spacefill atom and a docked farnesyldiphosphate is shown as a stick representation (red). (6.59 MB TIF) [file pone.0010760.s003.tif]
